# Comparing two different automatic methods to measure femoral neck-shaft angle based on PointNet++ network

**DOI:** 10.1038/s41598-022-16695-1

**Published:** 2022-07-20

**Authors:** Zhe Li, Jiayu Yang, Xinghua Li, Kunzheng Wang, Jungang Han, Pei Yang

**Affiliations:** 1 School of Computer, Xi’an University of Posts and Telecommunications, Xi’an, 710121 Shaanxi People’s Republic of China; 2 Department of Bone and Joint Surgery, The Second Affiliated Hospital of Medical College, Xi’an Jiaotong University, Xi’an, 710004 Shaanxi People’s Republic of China; 3 Department of Radiology, The Second Affiliated Hospital of Medical College, Xi’an Jiaotong University, Xi’an, 710004 Shaanxi People’s Republic of China

**Keywords:** Machine learning, Bone

## Abstract

Accurate measurement of the femoral neck-shaft angle (NSA) is of great significance for diagnosing hip joint diseases and preoperative planning of total hip arthroplasty. However, the repeatability of manual measurements is not as satisfactory, and the difference between 2D and 3D measurements is not clear. The computer-aided method provides a platform for automatic and accurate measurement of the NSA. The femoral point cloud datasets from 310 subjects were segmented into three regions, including the femoral head, femoral neck, and femoral shaft using PointNet++. We created a projection plane to simulate the hip anteroposterior radiograph and fitted the femoral neck axis and femoral shaft axis to complete the 2D measurement, while we directly fitted the two axes in space to complete the 3D measurement. Also, we conducted the manual measurement of the NSA. We verified the accuracy of the segmentation and compared the results of the two automatic and manual methods. The Dice coefficient of femoral segmentation reached 0.9746, and MIoU of that was 0.9165. No significant difference was found between any two of the three methods. While comparing the 2D and 3D methods, the average accuracy was 98.00%, and the average error was 2.58°. This paper proposed two accurate and automatic methods to measure the NSA based on a 2D plane and a 3D model respectively. Although the femoral neck and femoral shaft axes did not intersect in 3D space, the NSAs obtained by 2D and 3D methods were basically consistent.

## Introduction

The femoral neck-shaft angle (NSA) is a critical parameter of the proximal femur, defined as the angle formed by the longitudinal axis of the femoral neck and that of the femoral shaft. The biological significance of the NSA is to optimize the contact area between the femoral head and the acetabulum, as well as lower limb alignment^1,2^. It is further utilized to assess the anatomy of the proximal femur and guide the application of the hip prostheses^3–5^. Total knee arthroplasty (THA) often requires precise pre-operative planning for the NSA reconstruction of the complex hip^6^. In addition, hip prostheses with different NSA and femoral offset have been developed in recent years, requiring pre-operative NSA planning for patients. Therefore, accurate measurement of NSA is of great significance for THA. However, the literature indicated that the subjective factors of doctors affected the measurement results greatly^8^. It was reported that the manual measurement error was between 3.9º and 10.3º, and the intra-observer correlation coefficients (ICC) of manual measurement results ranged from 0.76 to 0.95 and the inter-observer ICC of those from 0.58 to 0.89, which indicated that the repeatability of manual methods was not as satisfactory^6,8^. Thus, computer-aided automatic measurement of NSA could achieve high repeatability to solve this problem effectively. Furthermore, with the development of surgical robots in recent years, the application of CT data for preoperative planning is popular. Different from the traditional method of 2-dimensional (2D) X-ray measurement, CT data can be used to achieve 3-dimensional (3D) measurement of the NSA easily. Taken together, the automatic and accurate measurement of NSA using CT data is of great significance for THA, and has a wide application value in the preoperative planning of surgical robotics.

At present, clinicians often use the hip anteroposterior radiography to measure the NSA, which requires the patient to lie supine on the photographic table with lower limbs straight, toe tips slightly inward rotation 20° to contact the two toes and heel separation^9^. However, the 2D image cannot accurately reflect the whole structure of the femur. Different anteversion angles of the femur, non-standard shooting position, and the scaling effect of varying equipment could interfere with the measurement results, resulting in an error of up to 12%^10^. Many studies reported 3D measurement based on CT and MRI data^11–14^, but to our knowledge, we found that the fitting line of the femoral neck and femoral shaft did not always intersect in 3D space. Therefore, we hypothesized a significant difference between the results obtained by directly applying the two fitting lines in the 3D model and simulating the original concept of measuring the NSA in a 2D image. Based on the above problems and hypothesis, this study applied CT data, developed two different automatic methods to measure NSA in 3D model and 2D image respectively, compared the significant difference between the two results, and verified the accuracy by comparing the two results with those measured manually by doctors.

The main contributions of this paper are as follows: 1. We proposed two different automatic methods to measure the NSA on CT data. We verified the effectiveness and accuracy of the two methods by comparing their results with manual measurement results by the doctor. 2. We used PointNet++ network to segment the femoral shaft, femoral neck, and femoral head with high precision, providing conditions for accurate measurement. The femoral neck and shaft axes could be accurately fitted by using the mathematical features of the femur shape. 3. We restored the original definition of a hip anteroposterior radiograph by projecting the 3D reconstruction model of the femur.

## Materials and methods

**Figure 1:**
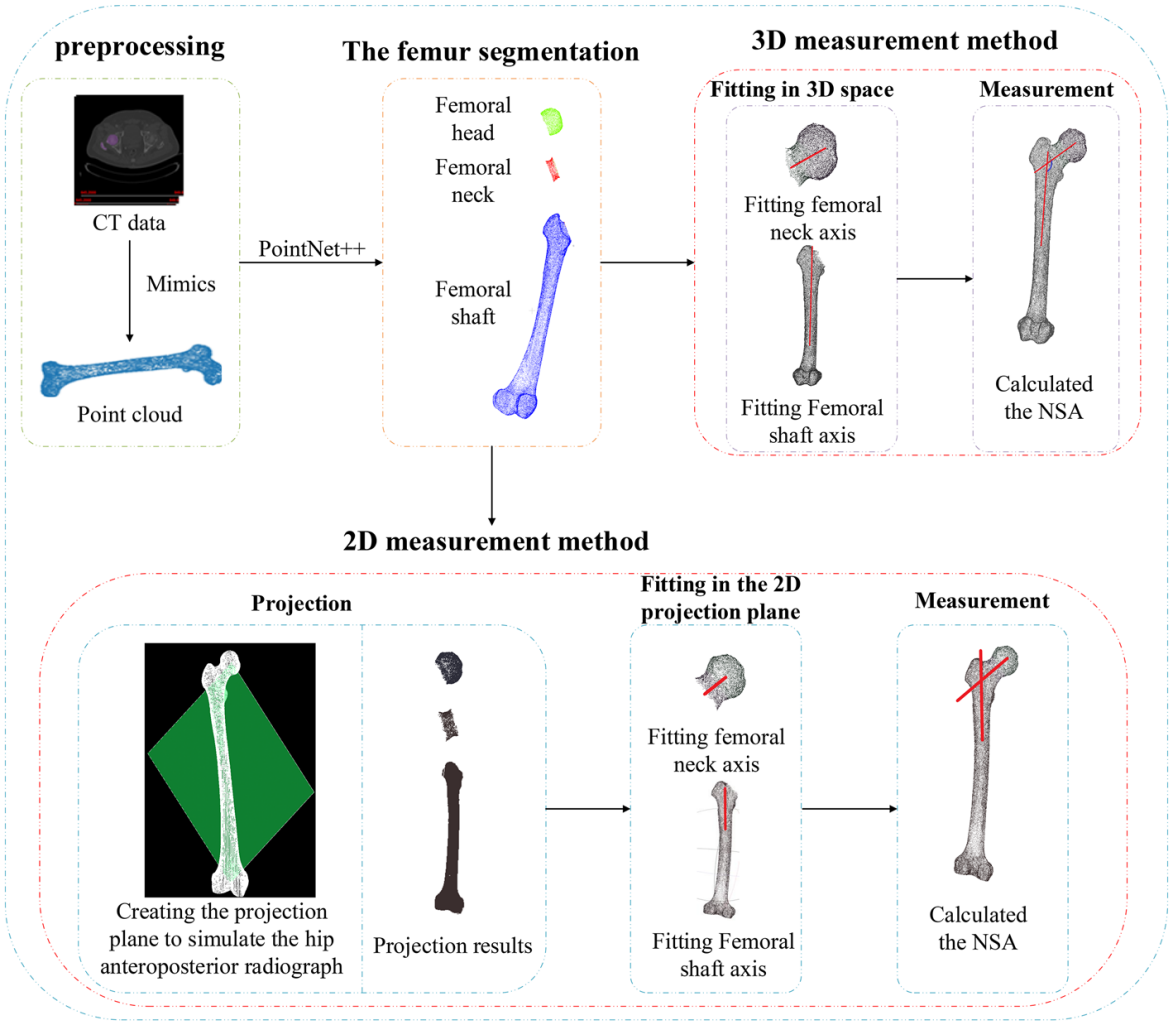
Automatic measurement process diagram.

This article proposed two automatic methods to measure the NSA, as shown in Fig. 1. First, we performed the 3D reconstruction of the femoral model from the original CT data to obtain the 3D femoral point cloud data. Then, we used the PointNet++ network to segment the femur into the femoral head, femoral neck, and femoral shaft and applied mathematical features to fit the femoral neck axis and femoral shaft axis. Finally, we measured the NSA in 2D and 3D, respectively. In the 2D method, we found the optimal projection plane to simulate the hip anteroposterior radiograph, projected the segmented models onto the plane, and used mathematical features to fit the femoral neck and shaft axes. While in the 3D method, we used directly mathematical features to fit the femoral neck and shaft axes and calculated the NSA. Also, we performed manual measurements of the NSA and compared the results of these three methods.

### Data collection

The CT data of 310 subjects from the Second Affiliated Hospital of Xi'an Jiaotong University were collected retrospectively from January 2017 to May 2021. The local ethics committee of the Second Affiliated Hospital of Xi'an Jiaotong University approved the project (number: 2022009) for this retrospective study on CT data. This study was conducted in accordance with the relevant guidelines and regulations with informed consent obtained from all subjects. The investigation followed the guidelines of the Helsinki Convention. Using a CT scanner (GE revolution CT, General Electric Company, Milwaukee, Wis), we collected CT datasets with 0.625*0.625 mm voxels and reconstruction thickness 1 mm. Only the intact femurs were included, and any diseases affecting the femur shape such as fracture, bone defect, bone tumor were excluded. We used the regional growth and mesh reconstruction steps in Mimics software (Materialise Inc., Leuven, Belgium) to perform the 3D reconstruction of the femur. And each reconstructed femur model was exported in the point cloud data format.

### Femur segmentation based on PointNet++

PointNet++ is a neural network model proposed by Charles et al. in 2017^15^, which is mainly used for point cloud segmentation and classification. This study used the segmentation module of PointNet++ to segment the femoral head, femoral neck, and femoral shaft. We divided the point cloud datasets into two parts: 50 femur data segmented manually by doctor were used to train the neural network, and the data of the remaining 260 femurs were used as test data. Cross-validation was adopted in the training, with 80% of the training data as the training set and 20% as the verification set.

Point cloud data can be expressed as n*3 matrix and has the characteristics of the disorder, which means that the arrangement order of each point in the point cloud does not affect its representation of the overall structure and shape in space. Most of the processing methods of the point cloud are to map the data into a two-dimensional image group or rasterize to perform subsequent processing. Still, the processing method often causes complicated calculations. The PointNet neural network model can take the point cloud data directly as the network's input and extract the point cloud features, such as distance and category, which is a point-cloud-based 3D deep learning network proposed by Charles et al. at Stanford University in 2017^16^. The PointNet network model uses s a single max-pooling operation to aggregate the whole point set. Since the output of the symmetric function does not change with the order of the input factors, all points in the point cloud set can be processed by the symmetric function to obtain the global characteristics of the point cloud set. However, when the local features are processed, and the point cloud density varies greatly, the processing effect of PointNet is not ideal. Charles et al. proposed a new neural network model based on PointNet and named it PointNet++ network to solve this problem^15^. The basis of the PointNet++ network is to divide the input point cloud into several overlapping regions at a certain distance in the metric space, extract the local features of the point cloud from each region, capture the geometric information, and repeat the whole process to get the feature set of the entire point cloud model. This process is similar to the convolutional neural network, which selects a specific convolution kernel size to extract the image's local features.

**Figure 2:**
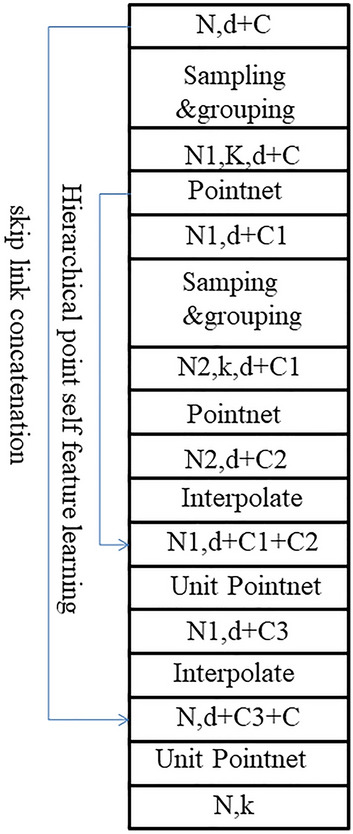
Network structure of PointNet++.

PointNet++, as a subsequent modification of PointNet, is essentially a layered version of PointNet, which can be used for point cloud segmentation and classification. This study used the segmentation network of PointNet++, as shown in Fig. 2. The segmentation process is to extract a global feature from the point cloud and gradually up-sample through this global feature. When up-sampling, Broadcasting replication is adopted, which means that the characteristics of the points near each point become the same as this point. However, this method will make it impossible to deal with some conflicting points or points that are not covered by the range. The farther the point is, the smaller its weight is. To achieve a better classification, we applied the linear variance to make a global normalization of the weight of each point, and the specific formula is as follows:

1 $$\begin{gathered} {\text{U}}\left( {\text{x}} \right) = \left\{ {\begin{array}{*{20}c} {\frac{{\mathop \sum \nolimits_{{{\text{i}} = 1}}^{{\text{N}}} {\text{w}}_{{\text{i}}} \left( {\text{x}} \right){\text{u}}_{{\text{i}}} { }}}{{\mathop \sum \nolimits_{{{\text{i}} = 1}}^{{\text{N}}} {\text{w}}_{{\text{i}}} \left( {\text{x}} \right)}},\,\,if\,\, d\left( {{\text{x}},x_{i} } \right) \ne 0 \quad for \,\,all \,\,in \,i } \\ { {\text{u}}_{{\text{i}}} , if \,d\left( {{\text{x}},x_{i} } \right) \ne 0\quad for\,\, all\,\, in\, i.} \\ \end{array} } \right. \hfill \\ {\text{where}}\,\,\,{\text{w}}_{{\text{i}}} \left( {\text{x}} \right) = { }\frac{1}{{{\text{d}}\left( {{\text{x}},{\text{x}}_{{\text{i}}} } \right)^{{\text{p}}} }},\,\,\,{\text{j }} = { }1,2,{ } \ldots ,{\text{C }} \hfill \\ \end{gathered}$$

The training environment of this model was as follows: python 3.6, pytorch1.1.0, cuda9.0, on a DGX-1 server with 8 Tesla V100 GPUs. The Adam optimization method is used for gradient descent (β1 = 0.9, β2 = 0.99), the learning rate is 0.01, batch size = 128, epoch = 1000, and numpoint = 2048.

The cross entropy loss function we used is as follows:

2 $$\mathrm{L}= \frac{\mathrm{i}}{\mathrm{N}}\sum_{\mathrm{i}}{\mathrm{L}}_{\mathrm{i}}=\frac{1}{\mathrm{N}}\sum_{\mathrm{i}}-\sum_{\mathrm{c}=1}^{\mathrm{M}}{\mathrm{y}}_{\mathrm{ic}}\mathrm{log}({\mathrm{P}}_{\mathrm{ic}})$$

where M is the number of categories, c represents one of the categories in M, and N represents the number of samples. ${\mathrm{y}}_{\mathrm{ic}}$ is a binary value that represents whether the predicted value is correct, and ${\mathrm{p}}_{\mathrm{ic}}$ represents the predicted probability that the sample i belongs to category c.

### 2D measurement of the NSA

#### Projection

According to the requirements of the hip anteroposterior radiograph, internal rotation of the lower limb counterbalances the effect of the femoral anteversion and keeps the femoral neck axis the longest. The purpose of our 2D method was to simulate the standard position of the hip anteroposterior radiograph. Compared with sagittal plane reported in the literarure, our projection method counterbalanced the femoral anteversion and was more sophisticated^17^. We used the following method to determine the projection plane: first, we segmented the femoral head, femoral neck, and femoral shaft using the Pointnet++ network and extracted 10% of the distal part of the femoral shaft. Next, as a sphere-like object, the femoral head was fitted to a spherical surface by the least square method to obtain the center coordinates. The calculation formula is as follows:

3 $$\begin{gathered} \left( {\sum {\text{u}}_{{\text{i}}}^{2} } \right){\text{u}}_{0} + \left( {\sum {\text{u}}_{{\text{i}}} {\text{v}}_{{\text{i}}} } \right){\text{v}}_{0} + \left( {\sum {\text{u}}_{{\text{i}}} {\text{w}}_{{\text{i}}} } \right){\text{w}}_{0} = { }\frac{{\sum \left( {{\text{u}}_{{\text{i}}}^{3} + {\text{u}}_{{\text{i}}} {\text{vi}}_{{\text{i}}}^{2} + {\text{u}}_{{\text{i}}} {\text{w}}_{{\text{i}}}^{2} } \right)}}{2} \hfill \\ \left( {\sum {\text{u}}_{{\text{i}}} {\text{v}}_{{\text{i}}} } \right){\text{u}}_{0} + \left( {\sum {\text{v}}_{{\text{i}}}^{2} } \right){\text{v}}_{0} + \left( {\sum {\text{v}}_{{\text{i}}} {\text{w}}_{{\text{i}}} } \right){\text{w}}_{0} = { }\frac{{\sum \left( {{\text{u}}_{{\text{i}}}^{2} {\text{v}}_{{\text{i}}} + {\text{v}}_{{\text{i}}}^{3} + {\text{v}}_{{\text{i}}} {\text{w}}_{{\text{i}}}^{2} } \right)}}{2} \hfill \\ \left( {\sum {\text{u}}_{{\text{i}}} {\text{w}}_{{\text{i}}} } \right){\text{u}}_{0} + \left( {\sum {\text{v}}_{{\text{i}}} {\text{w}}_{{\text{i}}} } \right){\text{v}}_{0} + \left( {\sum {\text{w}}_{{\text{i}}}^{2} } \right){\text{w}}_{0} = { }\frac{{\sum \left( {{\text{u}}_{{\text{i}}}^{2} {\text{w}}_{{\text{i}}} + {\text{vi}}_{{\text{i}}}^{2} {\text{w}}_{{\text{i}}} + {\text{w}}_{{\text{i}}}^{3} } \right)}}{2} \hfill \\ \end{gathered}$$

4 $$\mathrm{R}= \surd \frac{{\sum }_{\mathrm{i}=1}^{\mathrm{N}}({\left({\mathrm{u}}_{\mathrm{i}}-{\mathrm{u}}_{0}\right)}^{2}+{\left({\mathrm{v}}_{\mathrm{i}}-{\mathrm{v}}_{0}\right)}^{2}+{({\mathrm{w}}_{\mathrm{i}}-{\mathrm{w}}_{0})}^{2})}{\mathrm{N}}$$

5 $${\text{Where}}\,\,\,{\text{u}}_{{\text{i}}} = {\text{x}}_{{\text{i}}} - \overline{x}{\text{ v}}_{{\text{i}}} = {\text{y}}_{{\text{i}}} - \overline{y}{\text{ w}}_{{\text{i}}} = {\text{z}}_{{\text{i}}} - \overline{z}{\text{, i }} = \, 0, \ldots ,{\text{N}}$$

**Figure 3:**
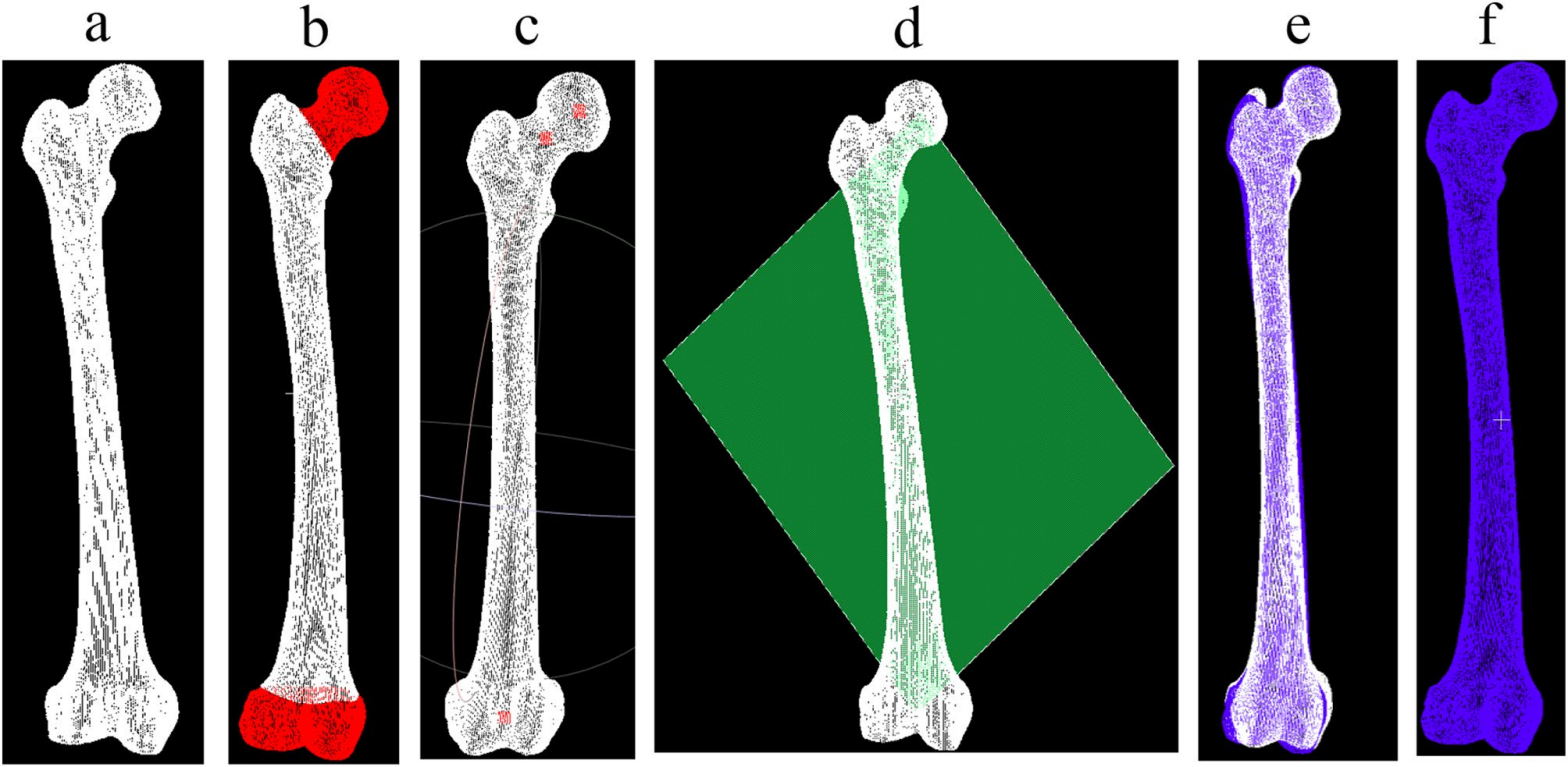
Projection plane diagram. (**a**) The point cloud model. (**b**) Segmenting the femoral head, femoral neck and 10% of the distal part of femoral shaft. (**c**) Calculating the centroids of the femoral head, femoral neck and 10% of the distal part of femoral shaft. (**d**) Determining the projection plane through the three centroids. (**e**) Projecting the femur to the projection plane. (**f**) The projection result.

The centroids of the femoral neck and 10% of the distal femur were also calculated. Finally, the three points were used to determine the projection plane where the 2D automatic measurement of the NSA was performed. It is shown in Fig. 3. The projection principle of this method used three dimensional plane equations in space as follows:

6 $$\mathrm{Ax}+\mathrm{By}+\mathrm{Cz}+\mathrm{D }= 0$$

The vertical foot of any point Vi in the point cloud to the plane is denoted as Vi '(x,y,z), and the line ViVi' is parallel to the normal vector of the plane. The parametric equation of line ViVi' is as follows:

7 $$\begin{gathered} {\text{x }} = {\text{ x}}_{{\text{i}}} { } - {\text{At}} \hfill \\ {\text{y }} = {\text{ y}}_{{\text{i}}} { } - {\text{At }} \hfill \\ {\text{z}} = {\text{ z}}_{{\text{i}}} { } - {\text{At }} \hfill \\ {\text{Where}}\,\,{\text{t }} = { }\frac{{{\text{Ax}}_{{\text{i}}} + {\text{By}}_{{\text{i}}} + {\text{Cz}}_{{\text{i}}} + {\text{D}}}}{{{\text{A}}^{2} + {\text{B}}^{2} + {\text{C}}^{2} }} \hfill \\ \end{gathered}$$

#### Femoral neck axis fitting

This study used the PointNet++ network to segment the femoral regions and projected a femoral model to the projection plane. Next, we used Random sample consensus (RANSAC) to obtain the center of the sphere of the projected femoral head and the centroid of the femoral neck, which formed the femoral neck axis^18^. The basic process of the RANSAC is as follows:

1. The minimum data set that could estimate the model was randomly selected from the input data, and the corresponding model parameters were calculated using the data set.

2. We Substituted all the data into the model and calculated outliers and inliers. Data exceeding the error threshold were considered outliers, while data less than the error threshold was considered inliers.

3. The number of repetitions of steps 1–2 was related to the outliers proportion and confidence of the model. The calculation process is shown in Eq. (8).

8 $$\mathrm{S }= \frac{\mathrm{log}(1-\mathrm{P})}{\mathrm{log}(1-{\uprho }^{\mathrm{K}}}$$

S is the number of minimum tests required, *P* is the confidence, p is the percentage of inliers, and *K* is the number of random samples.

Due to the irregular shape of the femoral neck, we solved the centroid of the femoral neck, and its formula is as follows:

9 $$\mathrm{pc}= \frac{1}{\mathrm{n}}\left(\sum_{0}^{\mathrm{n}}{\mathrm{x}}_{\mathrm{i}},\sum_{0}^{\mathrm{n}}{\mathrm{y}}_{\mathrm{i}},\sum_{0}^{\mathrm{n}}{\mathrm{z}}_{\mathrm{i}}\right)$$

where $\mathrm{i }= 0,....,\mathrm{C}$. C and n are the number of points.

#### Femoral shaft fitting

We used the projected femoral shaft and connected the centroid of 60% ~ 70% of the total length of the femoral shaft and the centroid of 70% ~ 80% of that as the femoral shaft axis.

#### Calculation of the NSA

While obtaining the femoral neck axis and the femoral shaft axis, we used the 3D vector angle formula to calculate the NSA. The calculation formula is as follows:

10 $$\begin{gathered} \cos \theta = \frac{A \times B}{{\left| A \right| \times \left| B \right|}} \hfill \\ {\uptheta } = \arccos {\uptheta } \hfill \\ \end{gathered}$$

where A is the vector representing the femoral neck axis, and B is vector representing the femoral shaft axis.

### 3D measurement of the NSA

We connected the spherical center of the femoral head and the centroid of the femoral neck obtained by the above steps as the femoral neck axis. We defined the line in space connecting centroid of 60–70% of the total length of the femoral shaft and the centroid of 70–80% of that as the femoral shaft axis. We finally calculated the NSA using the 3D vector angle formula same as the 2D method.

In terms of the software in 2D and 3D methods, we used the Visual Studio (version 2015, Microsoft, Inc, Seattle, Washington, USA) and Point Cloud Library (version 1.8.1) to calculate the NSA, and applied cloud compare (version 2.11, alpha, Anoia) to implement visualization.

### Manual measurement of the NSA

**Figure 4:**
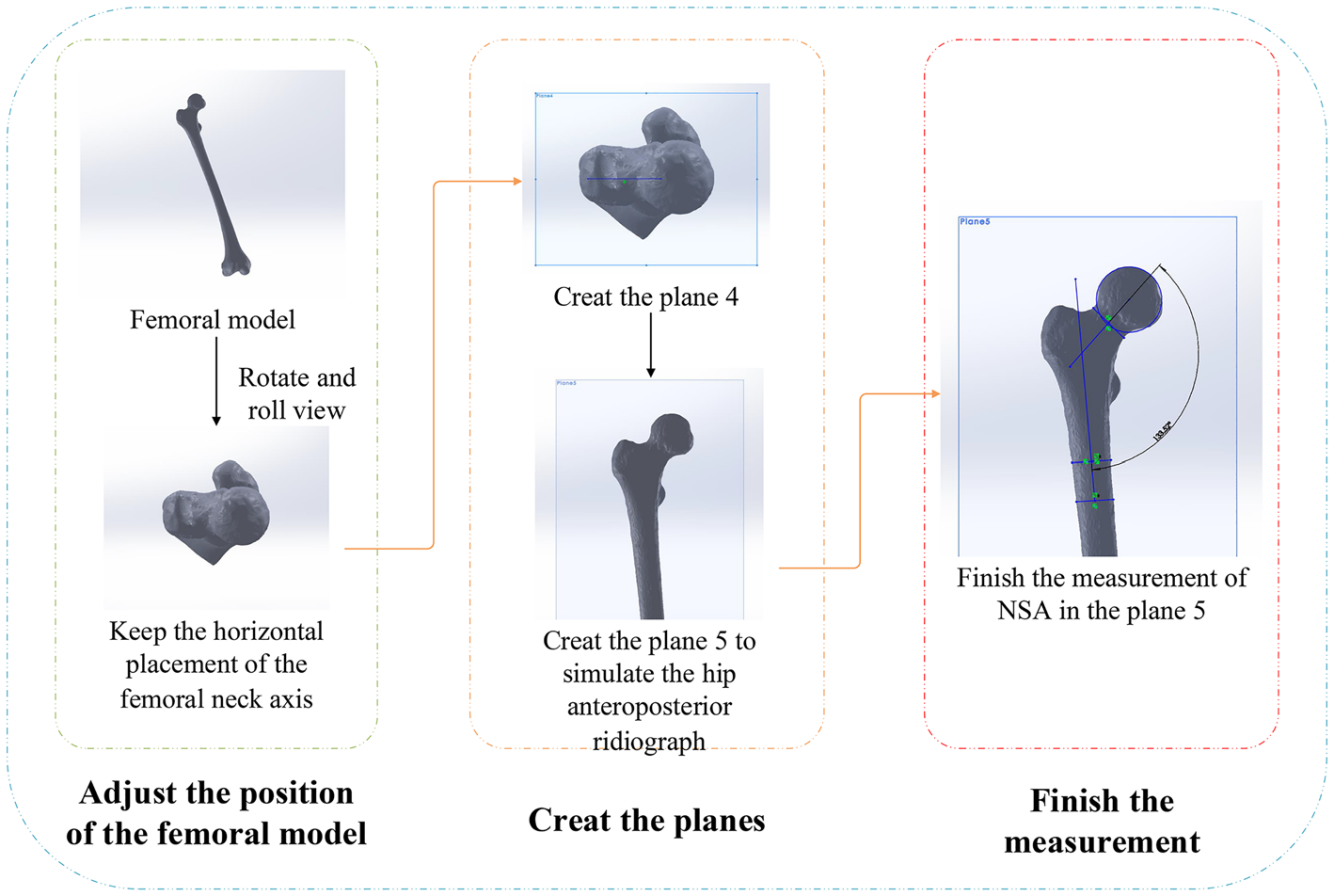
Manual measurement diagram.

We used the Solidworks software (version 2017, Dassault Systemes Company, Concord, 121 Massachusetts) to achieve the manual measurement. Firstly, we imported the femoral model and adjusted the position of the model to observe from the femoral head to knee with the overlap of the greater trochanter and the posterolateral condyle of the knee and the horizontal placement of the femoral neck axis. Next, we generated the plane 4 parallel to the screen, drew the femoral neck axis on the 2D plane 4, and created the new plane 5 perpendicular to the plane 4 with passing the femoral neck axis. Finally, we achieved the measurement on the 2D plane 5. We defined the femoral neck axis as the line connecting the center of the circle fitting the femoral head with the midpoint of the transverse axis of the femoral neck and the femoral shaft axis as the line connecting the two midpoints of the two transverse axes on the proximal femoral shaft. The NSA was the intersection angle between the two axes, which was shown in Fig. 4.

### Evaluation of the results

To accurately evaluate the segmentation effect of this algorithm on the femoral regions, The Dice coefficient and Mean Intersection over Union (MIoU) index were used to evaluate the performance of the segmentation. Dice coefficient is a set similarity measurement function, which is usually used to calculate the similarity of two samples. MIoU is the average of all IOUs, and IOU is the ratio of the intersection and union of the predicted and true values. The calculation formula is as follows:

11 $$\begin{gathered} {\text{Dice}} = { }\frac{{2\left| {{\text{x}} \cap {\text{y}}} \right|}}{{\left| {\text{x}} \right| + \left| {\text{y}} \right|}} \hfill \\ {\text{MIoU}} = { }\frac{1}{{{\text{k}} + 1}}\mathop \sum \limits_{{{\text{i}} = 0}}^{{\text{k}}} \frac{{{\text{TP}}}}{{{\text{FN}} + {\text{FP}} + {\text{TP}}}} \hfill \\ \end{gathered}$$

TP: the prediction is correct, the prediction result is positive, and the truth is positive. FP: the prediction is false, the prediction result is positive, and the truth is negative. FN: the prediction is false, the prediction result is negative, and the truth is positive. TN: the prediction is correct, the prediction result is negative, and the truth is negative. X is the segmentation result set output by the algorithm, and Y is the segmentation result set manually segmented by the doctor.

To evaluate the accuracy of the two automatic methods and analyze the consistence between the 2D and 3D methods, we calculated the error and average accuracy. The formulas are calculated as follows:

12 $$\begin{gathered} {\text{error}} = \left| {{\text{NSA}}_{{\text{m}}} - {\text{NSA}}_{{\text{a}}} } \right| \hfill \\ \overline{{{\text{error}}}} { } = { }\frac{{\mathop \sum \nolimits_{{{\text{i}} = 1}}^{{\text{n}}} {\text{error}}}}{{\text{n}}} \hfill \\ \end{gathered}$$

13 $$\begin{gathered} {\text{Accuracy}} = \left( {1 - \left| {\frac{{{\text{NSA}}_{{\text{m}}} - {\text{NSA}}_{{\text{a}}} }}{{{\text{NSA}}_{{\text{m}}} }}} \right|} \right) \times 100{\text{\% }} \hfill \\ \overline{{{\text{Accuracy}}}} { } = { }\frac{{\mathop \sum \nolimits_{{{\text{i}} = 1}}^{{\text{n}}} {\text{Accuracy}}}}{{\text{n}}} \hfill \\ \end{gathered}$$

Also, we used the two-sample Student’s t-test to determine the significance of the difference between any two of three methods. All statistical analyses including the Dice coefficient, MIoU index, average error, average accuracy, and t test were completed in Python version 3.6. And P values less than 0.05 were considered significant.

## Results

### Accuracy of the segmentation

The Dice coefficient of femoral segmentation reached 0.9746, indicating that the results of femoral segmentation were highly similar to the results of manual segmentation. MIoU was 0.9165, which proved that the robustness and accuracy of the method were high and showed that the PointNet++ network model could achieve high-precision segmentation of the femoral regions.

### Measurement results of the NSA

This study proposed two different automatic methods to measure the NSA. The measurement results of the two automatic methods and manual methods are shown in Table 1. The results showed that no significant difference was found between any two of three methods.

**Table 1:** Measurement results of the NSA.

	Manual results	2D automatic results	3D automatic results	Error.a	Error.b	Error.c
Degree (°)	126.76 ± 4.859 (114.04–139.90)	126.90 ± 4.980 (112.88–139.13)	126.88 ± 4.846 (113.08–139.82)	1.68 ± 1.10 (0.01–4.99)	2.71 ± 1.04 (0.24–5.10)	2.58 ± 1.74 (0.02–7.34)
P value	–	–	–	0.86	0.88	0.98
Average accuracy	–	–	–	98.67%	97.86%	98.00%

Data are presented as mean ± SD. Maximum and minimum values are indicated in brackets.

a represents the comparison between manual results and 2D automatic results. b represents the comparison between manual results and 3D automatic results. c represents the comparison between 2D automatic results and 3D automatic results. a, b, and c represents the same meanings in terms of the error, P value, and accuracy.

P value was calculated by t test between any two of three measurement results.

**Figure 5:**
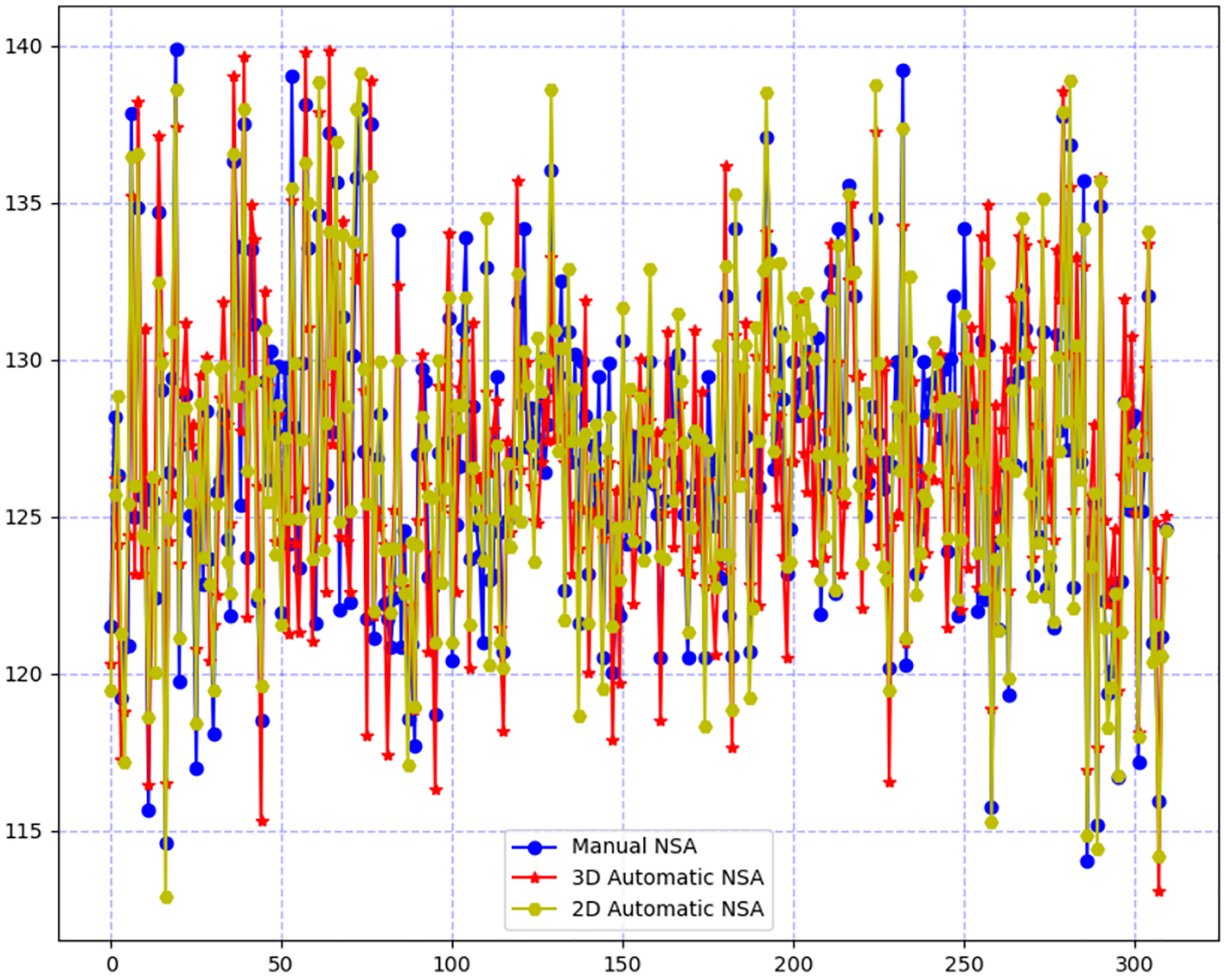
Comparison of the measurement results of the two automatic methods and manual method.

Comparison of the measurement results of the two automatic methods and manual methods is shown in Fig. 5, which showed that the three outcomes were consistent with a significant overlap area. Compared with the manual measurement results, the average error of 3D automatic measurement was 2.71° with an average accuracy of 97.86%, while the average error of 2D automatic measurement was 1.68° with an average accuracy of 98.67%. It showed that the 2D results were more closely equivalent to the manual results. While comparing the 2D and 3D methods, the average error was 2.58° with an average accuracy of 98.00%, which indicated that although no significant difference was found between the two automatic methods, the average difference of 2.58° existed. These results are also shown in Table 1.

## Discussion

This paper proposed two automatic methods to measure the NSA based on CT data: the 2D measurement by projecting the 3D model to the 2D projection plane and direct 3D measurement. First, we used PointNet++ neural network to achieve high-precision segmentation of the femoral regions. Next, we used the least-squares method to fit the femoral head area to obtain the spherical center coordinates, which transformed the regression problem of the spherical center coordinates of the femoral head to a segmentation problem of the femoral head region, simplified the complexity of the whole issue, and reduced the fitting error of the spherical center of the femoral head. We further obtained the centroids of the femoral neck and the distal femur to create the projection plane and projected models to the plane. Based on the mathematical characteristics of the femoral shape, the femoral neck axis and femoral shaft axis were calculated using the centroids in the projection plane and 3D, respectively. Finally, we finished the 2D and 3D measurements of the NSA separately.

We verified the accuracy of our methods by comparing the two automatic results with manual results, which both showed high accuracy. Since the basic concept of the 2D automatic measurement method was the same as the manual method, the accuracy of the 2D automatic method was higher than the 3D method. By comparing the results of 2D and 3D automatic methods, we found no statistically significant difference between them. Therefore, in clinical application, we thought that it could be approximated that the 3D CT measurement results of NSA were consistent with the measurement results from the standard hip anteroposterior radiograph. We could measure the NSA with standard X-ray measurement techniques in preoperative planning and directly match it with the hip prosthesis. Suppose the hip anteroposterior radiography could be shot in a standard position to eliminate the femoral anteversion effect. In that case, it is not necessary for all patients to undergo a CT examination with higher costs and more rays. However, this study still had an average error of 2.58° and a maximum error of 7.34° between the two automatic methods. And there was a large difference between the maximum error and the minimum error. Except for the systematic error, we believed it might be caused by individual differences in the distance between the femoral neck and the femoral shaft axes in space. Thus, when dealing with complex situations requiring more precise manipulation, we might need to evaluate more parameters than the NSA comprehensively. Further clinical experiments were needed to confirm the above clinical applications. Besides, when different manual measurement results of the NSA were reported in different studies^5,19–21^, we believed that manual measurement was somewhat subjective. In contrast, the automatic measurement could provide better repeatability and accuracy, which also needed to be verified by further clinical experiments.

When we consider the integration of automatic measurement of NSA into clinical applications such as surgical robots, the two automatic measurement methods proposed in this study had their own advantages and disadvantages. We believed that the 3D measurement method was more straightforward, but the results varied due to the different distance between the femoral neck axis and the femoral shaft axis, which cannot be described by the NSA. In 2D measurement, the two axes were projected on the same standard plane and the two axes intersected, which solved the problem of disjunction of the two axes to some extent. However, the result might be inaccurate due to the difference of the distance and tilt between the two axes in the projection process. None of them solved the inconsistent problem of measurement results caused by disjunction of two axes. Thus in our further study, we will apply statistical shape model to study the morphological variation of the proximal femur and explore a better parameter or the fusion of multiple parameters to describe the femoral neck and femoral morphological characteristics. Including more anatomical information enables a more accurate description of the proximal femur shape, guides the selection of the prosthesis for preoperative planning, and assisted in improved prosthesis design.

This study utilized the line formed by centroids of 60% ~ 70% of the total length of the femoral shaft and 70% ~ 80% of that as the femoral shaft axis. Pulkkinen et al. reported in the hip anteroposterior radiograph that the midpoint of the transverse axis of the femoral shaft 3 cm below the lesser trochanter was used as a reference for the femoral shaft axis^22^. However, we found that the region near the lesser trochanter was prone to errors due to the different curvature between the inner and outer parts, so we chose the region far away from the lesser trochanter. Meanwhile, we still chose the proximal femur to determine the femoral shaft axis because there was an obvious radian in the middle and distal regions of the femoral shaft, which resulted in a different direction from the proximal femur. Hartel et al. used the line formed by the midpoints of the transverse axes of the femoral shafts at 25% and 35% of the total femur length as the femoral shaft axis^11^. In comparison, we used the centroids of 60–70% and 70–80% regions of the total femur length to fit the femoral shaft axis to reduce the error caused by the irregular surface of the femur and the different radians of the inner and outer parts.

Many pieces of literature reported the novel methods to measure the NSA. Wei et al. proposed an automatic method to measure the NSA on radiograph. With their method based on the 2D radiographs, our methods based on CT data made the automatic measurement of the NSA comprehensive^23^. Hartel et al. used a novel 3D modeling and analytical technology to measure the NSA based on CT data. But, their accuracy was not verified and the results ranged from 100.1° to 146.2°^11^. He et al. applied the 3D multiline shape of the femur to measure the NSA based on the CT data and eliminated the influence of the shooting position^24^. However, the interaction method they used was complex and not fully automated. Casciaro et al. achieved the automatic and repeatable method to measure the NSA using cylindrical fitting of the femoral shaft and femoral neck. Still, they did not obtain accurate results due to the irregularity of the femoral morphology^25^. Compared to their method, the centroids of the femoral regions might be a better way to eliminate errors caused by irregularities.

One of the limitations of this study was that corresponding X-ray data were not obtained. Although we created the standard hip anteroposterior projection to simulate the X-ray, such a precise alignment of the femur cannot be achieved in a clinical setting when generating planar X-rays. To obtain a more realistic clinical comparison result, we will collect the corresponding data of X-ray and CT data for further study and analysis in the future work.

## Conclusion

This paper proposed two accurate and automatic methods to measure the NSA based on a 2D plane and a 3D model. No significant difference between 2 and 3D measurement methods was found, and the average difference was 2.58°. Although the femoral neck and femoral shaft axes did not intersect in 3D space, the NSAs obtained by 2D and 3D methods were basically consistent. Either the 2D method on standard X-ray or the 3D method on CT could assist in selecting an appropriate hip prosthesis when making pre-operation planning for THA.

## Data Availability

The datasets used and/or analyzed during the current study available from the corresponding author on reasonable request.
